# Orlistat ameliorates lipid dysmetabolism in high-fat diet-induced mice via gut microbiota modulation

**DOI:** 10.3389/fmicb.2025.1480500

**Published:** 2025-02-06

**Authors:** Chengyan Huang, Yuanhui He, Ping Chai, Zongxin Liu, Sirui Su, Yanhui Zhang, Yuelan Luo, Shuiping Fu

**Affiliations:** ^1^Department of Medical Imaging, Fenyang College, Shanxi Medical University, Fengyang, China; ^2^Department of Obstetrics and Gynecology, Beijing Tongren Hospital Affiliated to Capital Medical University, Beijing, China; ^3^Department of Nursing, Fenyang College, Shanxi Medical University, Fengyang, China

**Keywords:** Orlistat, obesity, lipid metabolism, gut microbiota, microbiota transplantation

## Abstract

Orlistat reduces obesity by inhibiting gastrointestinal lipases, thereby blocking the absorption and accumulation of triglycerides in the intestine. It has been shown to improve lipid metabolism and alter intestinal microbial communities in animals and humans. However, the impact of Orlistat-induced changes in gut microbiota on obesity requires further investigation. In this study, we found that Orlistat significantly improved metabolic disorders, inhibited fat accumulation, and reshaped the structure of intestinal microbiota. Specifically, it reduced α diversity and increased the relative abundance of Verrucomicrobia and Akkermansia. Notably, antibiotic-induced gut microbiota depletion significantly weakened Orlistat’s effect on improving metabolic disorders. Furthermore, microbiota transplanted from Orlistat-treated mice effectively alleviated lipid metabolic disorders caused by a high-fat diet. We also observed that Orlistat increased food intake in mice and inhibited the synthesis of appetite-regulating hormones glucose-dependent insulinotropic polypeptide (GIP) and glucagon (Gcg). However, antibiotic-depleted microbiota mitigated this inhibitory effect. Interestingly, although Orlistat altered the gut microbiota of mice, transplanting these microbiota did not inhibit the synthesis of appetite-regulating hormones. In summary, our results suggest that Orlistat can reshape the gut microbiota, and the altered gut microbiota works synergistically with Orlistat to improve metabolic disorders. This improvement is related to the increased abundance of Verrucomicrobia and Akkermansia.

## 1 Introduction

The incidence of overweight and obesity is increasing worldwide, with nearly 2 billion adults classified as overweight and more than half of those considered obese (Hoffman et al., 2021). Growing evidence indicates that obesity is closely linked to chronic, low-grade inflammation, which can lead to various health issues including insulin resistance, type 2 diabetes, hypercholesterolemia, cardiovascular disease, obstructive sleep apnea, and multiple types of cancer (Everard et al., 2013; Ghaben and Scherer, 2019; Mayoral et al., 2020; Osborn and Olefsky, 2012; Lv et al., 2019). As a result, obesity prevention represents a major challenge for global public health.

The gut microbiota is a large and diverse group of microorganisms living in the human gastrointestinal tract. These microorganisms and their metabolites play an important role in regulating health and serve as a bridge between diet and host (Dalby et al., 2017). Additionally, the gut microbiota is influenced by the host’s birth method, lifestyle, genetics, and medication (Lynch and Pedersen, 2016). As early as 2005, research indicated that changes in the composition of the gut microbiota are associated with the development of obesity and its related metabolic disorders (Ridaura et al., 2013). An increased ratio of the major phyla *Firmicutes* to *Bacteroidetes*, along with changes in several bacterial species, can promote the development of obesity in dietary and genetic mouse models (Turnbaugh et al., 2006; Goodman et al., 2011). Studies in obese animals suggest that obesity-induced gut dysbiosis, caused by either a high-fat diet or genetic factors, impairs intestinal integrity (Cani et al., 2009; Cani et al., 2008; Caesar et al., 2015). This process leads to the release of the endotoxin lipopolysaccharide (LPS) from intestinal Gram-negative bacteria into the bloodstream (Saito et al., 2007). LPS, as an inflammatory trigger, combines with the Toll-like receptor 4 (TLR4) to activate the NF-κB signaling pathway and promotes the synthesis and release of inflammatory factors such as tumor necrosis factor-α (TNF-α), interleukin-6 (IL-6), and interleukin-1β (IL-1β) (Cani et al., 2008; Li et al., 2008). Increased levels of these inflammatory cytokines can lead to chronic low-grade systemic inflammation (CLGI) (Hotamisligil, 2006). Systemic CLGI damages pancreatic beta cells, disrupts insulin action, and mediates glucose intolerance in obesity, further aggravating metabolic disorders (Ewaschuk et al., 2007). Moreover, different gut microbiota compositions can influence host phenotypes through host-microbe metabolic axes. For example, gut microbiota from obese individuals can lead to obesity in germ-free mice (Ridaura et al., 2013).

Orlistat is an irreversible inhibitor of pancreatic and gastric lipases. It exerts its pharmacological activity by forming a covalent bond with the active serine site of gastric and pancreatic lipases in the lumen of the gastrointestinal tract. This action prevents these lipases from hydrolyzing dietary fat (in the form of triglycerides) into absorbable free fatty acids and glycerol (Hadváry et al., 1991; Borgström, 1988; Ransac et al., 1991; Heck et al., 2000). The unabsorbed triglycerides remain in the intestinal tract and are ultimately excreted through feces. Pharmacokinetic studies of Orlistat have shown that it is not appreciably absorbed into the systemic circulation and only exerts its pharmacological role in the intestinal tract (Heck et al., 2000; Zhi et al., 1995; Zhi et al., 1996). Orlistat demonstrated a good safety profile in a 4-year double-blind prospective study (Torgerson et al., 2004). An increasing number of studies have shown that gut microbiota is involved in the metabolism of many drugs and can affect their effectiveness (Zimmermann et al., 2019; Klünemann et al., 2021). For example, studies on statins, which regulate serum cholesterol and reduce the risk of heart disease, have shown that these drugs not only reduce cholesterol levels by inhibiting HMG-CoA reductase but also play a lipid-lowering role by interacting with gut microbiota (Nolan et al., 2017; Caparrós-Martín et al., 2017).

The gut microbiome is a critical component of digestion, responsible for breaking down complex carbohydrates, proteins, and fats. Microorganisms in the gut are known to express lipases, which can degrade triglycerides and phospholipids into their polar head groups and free lipids (Morales et al., 2016; Jaeger et al., 1994). Although only a very small proportion of total dietary fat reaches the colon (<5%) (Mu and Høy, 2004), it still plays a major role. However, microorganisms are thought to lack the ability to catabolize free lipids in the anaerobic environment of the gut (Cândido et al., 2018). These free lipids have antimicrobial properties and can directly interact with host pattern recognition receptors (Cândido et al., 2018; Desbois and Smith, 2010).

Degradation and absorption of dietary fat primarily occur in the small intestine, with little, if any, dietary fat reaching the colon in healthy individuals (Salonen and de Vos, 2014). The number of bacteria generally increases along the gastrointestinal tract, ranging from approximately ~10^8^ bacteria per gram (dry weight) of ileal contents to up to ~10^12^ bacteria per gram (dry weight) in the colon (Berg, 1996). Due to Orlistat’s lipase inhibition, triglycerides accumulate in the intestine, and unabsorbed triglycerides can reach the colon. However, free fatty acids exhibit potent antimicrobial effects at very small doses (Huang et al., 2011; Huang et al., 2010), suggesting that free fat reaching the colon considerably interacts with gut microbiota. Studies by Jin et al. (2022) and Ke et al. (2020), demonstrated that Orlistat exacerbates the reduction in microbial diversity and abundance caused by a high-fat diet (HFD), leading to decreased α diversity indices such as Chao1, Observed_OTUs, Shannon, and Simpson. Specifically, Jin’s et al. (2022) research revealed that Orlistat significantly enriched Actinobacteria and Proteobacteria, which are closely associated with lipid and glucose metabolism pathways. Meanwhile, Ke’s et al. (2020) study indicated that Orlistat increased the relative abundance of dominant bacteria like *Firmicutes* and *Akkermansia muciniphila* while decreasing *Desulfovibrio* and *Alistipes*. Additionally, Orlistat significantly increased fecal butyric acid levels while reducing acetic acid and propionic acid. Notably, *Akkermansia* was found to be negatively correlated with fasting blood glucose. These findings suggest that Orlistat not only mitigates obesity by directly inhibiting fat absorption but may also enhance metabolic health through modulation of the gut microbiome. However, despite extensive research on the effect of Orlistat on the gut microbiome, the direct relationship between Orlistat and reduced obesity requires further elucidation. Herein, we sought to investigate whether Orlistat-induced improvements in gut microbiota contribute to obesity reduction, thereby establishing a foundation for understanding the link between the Orlistat-induced increase in energy metabolism and gut microbiota.

## 2 Materials and methods

### 2.1 Animals and diet

All animal experiments reported in this manuscript were approved by the Ethics Committee for Scientific Research of Fenyang College of Shanxi Medical University (Approval No. 2023021). C57BL/6J male mice were purchased from Shanxi Medical University Laboratory Animal Central (Taiyuan, China), with permission code SCXK2019-0004. All animals were housed in individually ventilated cages during the experiment under the following conditions: ambient temperature of 20–26°C, relative humidity between 40–60%, minimum static pressure difference of 10 Pa, noise level ≤60 dB (A), and a 12-h light/dark cycle. Water and feed were provided *ad libitum*. The breeding cages for C57 mice measured 380 × 160 × 180 mm. At the end of the experiment, all mice were anesthetized with sodium pentobarbital and then euthanized by cervical dislocation.

Both the normal and HFDs used in the experiment were prepared manually in the laboratory. The main components and their proportions in the normal diet and the HFD are detailed in Table 1 (Anhê et al., 2019). The normal diet primarily consisted of whey protein, cornstarch, sucrose, and other components, while the high-fat diet significantly increased the fat content, particularly through the addition of lard and soybean oil.

**Table 1:** Food ingredient and content.

Ingredient	Normal diet		High-fat diet	
	g	kcal	g	kcal
Sucrose	100	400	200	800
Corn starch	527	2,108	177	708
Casein	200	800	200	800
L-cystine	3	12	3	12
Cellulose	55	0	55	0
Maltodextrin	35	140	35	140
Soybean oil	25	225	75	675
Lard	20	180	220	1980
NaCl	5	0	5	0
Calcium carbonate	15	0	15	0
Dicalcium phosphate	15	0	15	0
Potassium citrate	16.5	0	16.5	0
Mineral/vitamin mix	10	40	10	40
Total	1026.5	3,905	1026.5	5,155
	**g%**	**kcal%**	**g%**	**kcal%**
Protein	19.81	20	27.16	20
Fat	4.54	10	36.79	60
Carbohydrate	69.34	70	27.42	20
Available phosphorus	10	0	4	0
Lysine	4.8	0	12	0
Methionine	4	0	4	0
Total		100		100
(kcal/g)	3.80		5.02	

### 2.2 Orlistat treatment and Orlistat supplementation for antibiotic-treated mice

Thirty-six 8-week-old male C57BL/6J mice (21.3 ± 1.5 g) were randomly divided into four groups of nine mice each using a computer-based random number generator: normal diet (ND), high-fat diet (HFD), HFD + Orlistat (ORL), and antibiotic complex + ORL (Abx + ORL). The ND group was fed a normal diet, while the other three groups were fed a HFD. Mice in the ORL group received Orlistat (Zein Biotechnology, Chongqing, China) at 50 mg/kg/day. The drug was dissolved in normal saline and administered intragastrically at 200 μL per dose, once daily. Mice in the ORL + Abx group were treated similarly to the ORL group, with the addition of antibiotics (1 g/L metronidazole, 1 g/L ampicillin, 0.5 g/L neomycin, and 0.5 g/L vancomycin) in their drinking water (Secombe et al., 2021; Kennedy et al., 2018). All antibiotics were purchased from Sigma-Aldrich (Shanghai) Trading Co., Ltd., and drinking water was prepared daily. Each group was weighed and fed every Sunday morning at 9 am. After 10 weeks of Orlistat administration, blood samples were collected via orbital bleeding. Animals were then euthanized, and visceral adipose tissue (including epididymal and mesenteric fat) and subcutaneous adipose tissues were collected and weighed. Mice livers, jejunum, and caecum were also collected. The liver was fixed with 4% paraformaldehyde for 12 h for hematoxylin and eosin (H&E) staining, with the remaining liver tissue preserved at −80°C. Jejunum and caecum samples were rinsed with phosphate buffer solution (PBS, pH 7.3), then flash-frozen in liquid nitrogen and stored at −80°C for quantitative real-time reverse-transcription polymerase chain reaction (qRT-PCR) analysis.

### 2.3 Gut microbiota transplantation

Male mice aged 8 weeks were divided into four groups (*n* = 9 per group) using a computer-based random number generator: ND, HFD, high-fat diet mice fecal microbiota transplantation (FMT-HFD), and Orlistat-treated mice FMT (FMT-ORL). Prior to fecal microbiota transplantation, the FMT-HFD and FMT-ORL groups received antibiotics in their drinking water (ampicillin 0.5 g/L, neomycin 0.25 g/L, and metronidazole 0.5 g/L) (Secombe et al., 2021). Additionally, these groups were treated with 200 μL of an antibiotic cocktail (ampicillin 1 g/L, neomycin 0.5 g/L, vancomycin 0.5 g/L, and metronidazole 1 g/L) via oral gavage for 7 days to clear their gut microbiota. Following antibiotic treatment, three fecal samples were randomly collected from each cage of the FMT-HFD and FMT-ORL groups. Fecal samples were collected under a laminar flow hood to maintain a sterile environment. All equipment, including collection tubes and pipettes, was sterilized by autoclaving at 121°C for 20 min prior to use. The fecal suspension was prepared in a biosafety cabinet using sterile phosphate buffered saline (PBS, pH 7.3). The suspension was centrifuged at 3,000 rpm for 2 min, and the supernatant was collected under sterile conditions. Fresh transplant material was prepared immediately before each transplantation to avoid any potential microbial contamination. The fecal samples were thoroughly mixed and ground. Fecal bacterial solutions were then extracted by centrifugation at 3,000 rpm for 2 min. The bacterial content in the feces was estimated using the dilution plating method to assess the effectiveness of the antibiotic treatment. The samples were diluted 100-fold in sterile phosphate-buffered saline (PBS) and plated on LB solid medium (V900857, Sigma-Aldrich). The plates were incubated at 37°C for 24–48 h, and bacterial colonies were counted. The ND group, which did not receive antibiotic treatment, served as the control. The results confirmed a significant reduction in bacterial growth in the FMT-HFD and FMT-ORL groups compared to the ND group, indicating successful depletion of gut microbiota.

Eight-week-old male donor mice (*n* = 4 per diet group) were fed either a HFD or HFD supplemented with Orlistat for 15 weeks. After 4 weeks of feeding, stools were collected daily for the subsequent 2 weeks under sterile conditions in a laminar flow hood. Stools from donor mice of each diet group were pooled, and 200 mg was resuspended in 1 mL of PBS buffer solution. The mixture was centrifuged at 3,000 rpm for 2 min. The supernatant was collected and used as transplant material; this process took place in a biosafety cabinet. Fresh transplant material was prepared on the day of transplantation within 30 min before oral gavage to prevent changes in bacterial composition. The FMT-HFD and FMT-ORL groups were inoculated with fresh transplant material (200 μL per mouse) via oral gavage three times a week for 2 weeks (Borody et al., 2013). The mice were then euthanized for subsequent analysis.

### 2.4 Serum biochemical analysis

Serum samples were obtained by centrifuging blood at 3000 rpm for 10 min at 4°C. An Epoch microplate spectrophotometer was used to measure serum biochemical parameters. The biochemical assay kits used in this study were purchased from the Nanjing Jiancheng Bioengineering Institute (Nanjing, China). These include the Total Cholesterol Assay Kit (A111-1-1, single reagent GPO-PAP method), Triglyceride Assay Kit (A110-1-1, single reagent GPO-PAP method), High-Density Lipoprotein Cholesterol Assay Kit (A112-1-1, dual reagent direct method), Low-Density Lipoprotein Cholesterol Assay Kit (A113-1-1, dual reagent direct method), and Glucose Assay Kit (A154-1-1, glucose oxidase method). All kits were used strictly in accordance with the manufacturer’s instructions to ensure the accuracy and reliability of the results.

### 2.5 qRT-PCR

Total RNA from liver and small intestine was isolated from liquid nitrogen-frozen and ground tissues using RNAiso Plus (Takara 9109, China). Equal amounts of total RNA were used to synthesize cDNA with the PrimeScript^™^ RT reagent Kit with gDNA Eraser (Takara RR047A, China). Quantitative real-time reverse-transcription PCR (qRT-PCR) was performed in triplicate using TB Green, 96-well plates, and the CFX96 Real-Time PCR Detection System (Bio-Rad). Each well contained a total of 25 μL, consisting of 2 μL of cDNA, 2 μL of target primers, 8.5 μL of water, and 12.5 μL of TB Green Premix Ex Taq II. GAPDH was chosen as the housekeeping gene to normalize target gene levels. Hot-start PCR was performed for 40 cycles, with each cycle consisting of denaturation for 5 s at 95°C, followed by annealing and elongation for 30 s at 60°C. Relative quantification was done using the $2^{-\mathrm{\Delta \Delta CT}}$ method (Ke et al., 2020), where ΔΔCT = (CT Target − CT GAPDH) treatment − (CT Target − CT GAPDH) control. Relative expression was normalized and expressed as a ratio to the expression in the control group. The primers used are shown in Table 2.

**Table 2:** Gene primer sequence and their corresponding abbreviations: glyceraldehyde-3-phosphate dehydrogenase (*GAPDH*), interleukin-1β (*IL-1β*), interleukin-6 (*IL-6*), tumor necrosis factor-α (*TNF-α*), glucose-dependent insulinotropic polypeptide (*GIP*), glucagon gene (*Gcg*) and cholecystokinin (*CCK*).

	Forward	Reverse	Gene bank number
GAPDH	5′-GTGTTCCTACCCCCAATGTGT-3′	5′-ATTGTCATACCAGGAAATGAGCTT-3′	M32599
IL-1β	5′-TCCATGAGCTTTGTACAAGGA-3′	5′-AGCCCATACTTTAGGAAGACA-3′	NM_008361
IL-6	5′-GAGAGGAGACTTCACAGAGGATACC-3′	5′-TCATTTCCACGATTTCCCAGAGAAC-3′	NM_031168.2
TNF-α	5′-AGACCCTCACACTCAGATCA-3′	5′-TCTTTGAGATCCATGCCGTTG-3′	NM_013693
GIP	5′-TCGAGGTCCAAGGTACGCAGAG-3′	5′-CCAGCAGCCAGTTCACGAAGTC-3′	NM_008106
Gcg	5′-ACTCCCGCCGTGCCCAAG-3′	5′-TGCTGCCTGGCCCTCCAAG-3′	NM_008100
CCK	5′-GTGCGTGGTGATGGCAGTCC-3′	5′-CTCTTGCGCCCGCTGCTC-3′	NM_031161

### 2.6 H&E staining

Liver tissue samples preserved in 4% paraformaldehyde were dehydrated, cleared, and embedded in paraffin wax using a tissue processor. After solidification, the blocks were sectioned using a microtome to a thickness of 4–6 μm. The sections were mounted on glass slides and baked at 65°C for approximately 2 h. H&E staining was performed according to standard protocols (Cardiff et al., 2014).

### 2.7 Mice gut microbiota 16S rRNA gene sequencing

After sacrificing each group of mice, cecal contents were collected, flash-frozen in liquid nitrogen, stored on dry ice, and transported to Majorbio Bio-Pharm Technology Co. Ltd. (Shanghai, China) for analysis. Total DNA was extracted from the cecal content samples using the E.Z.N.A.^®^ soil DNA Kit (Omega Bio-tek, Norcross, GA, United States) according to the manufacturer’s instructions. DNA quality and concentration were assessed using a NanoDrop 2000 spectrophotometer (Thermo Fisher Scientific, Wilmington, DE, United States). Bacterial 16S rRNA gene fragments (V3–V4 region) were amplified from the extracted DNA using primers 338F (5′-ACTCCTACGGGAGGCAGCAG-3′) and 806R (5′-GGACTACHVGGGTWTCTAAT-3′). PCR conditions were as follows: 27 cycles of 95°C for 30 s, 55°C for 30 s, and 72°C for 45 s. Each PCR reaction (20 μL) contained 4 μL 5× TransStart FastPfu buffer, 2 μL 2.5 mM deoxynucleoside triphosphates (dNTPs), 0.8 μL of each primer (5 μM), 0.4 μL TransStart FastPfu DNA Polymerase, 10 ng of extracted DNA, and ddH_2_O to make up the final volume. Amplicon size was verified by agarose gel electrophoresis. Paired-end sequencing was performed on the Illumina MiSeq platform using PE300 chemistry at Majorbio Bio-Pharm Technology Co., Ltd. (Shanghai, China). Raw reads were deposited in the NCBI Sequence Read Archive database (Bioproject ID: PRJNA1130423).

After demultiplexing, the resulting sequences were merged using FLASH (v1.2.11) (Magoč and Salzberg, 2011) and quality-filtered with fastp (v0.19.6) (Chen et al., 2018). The high-quality sequences were then denoised using the DADA2 (Callahan et al., 2016) plugin in the Qiime2 (v2020.2) (Bolyen et al., 2019) pipeline using the recommended parameters, which allowed for single-nucleotide resolution based on error profiles within the samples. DADA2 denoised sequences are typically referred to as amplicon sequence variants (ASVs). Taxonomic assignment of ASVs was performed using the Naive Bayes consensus taxonomy classifier implemented in Qiime2 and the SILVA 16S rRNA database (v138). Analysis of the 16S rRNA microbiome sequencing data was performed using the free online platform of Majorbio Cloud Platform.^1^

### 2.8 Statistical analysis

Data are expressed as mean ± standard error of the mean (SEM). Differences between groups were assessed using unpaired two-tailed Student’s *t*-tests (GraphPad Prism 8 software). All statistical analyses were performed using one-way analysis of variance with homogeneity of variances tested via Levene’s test. A *p*-value of <0.05 was considered statistically significant.

## 3 Results

### 3.1 Antibiotics counteract Orlistat’s adipose-reducing properties in HFD-fed mice

**Figure 1:**
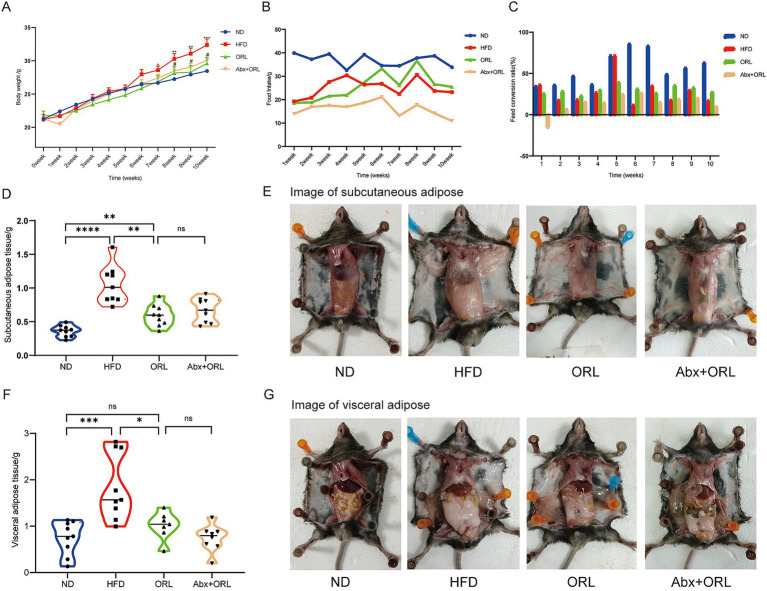
Orlistat treatment improved lipid metabolism in high fat diet fed mice. Changes in body weight (**A**, x ± s) and average food intake **(B)** in each group of mice (*n* = 9). **(C)** Feed conversion ratio = average food intake (g)/average weight gain (g) of each group of mice. Mice subcutaneous adipose tissue weight **(D)** and subcutaneous adipose tissue **(E)** appearances in animals in the ND, HFD, ORL and Abx + ORL groups. Appearance of visceral adipose tissue weight **(F)** and visceral adipose tissue **(G)** in animals in the ND, HFD, ORL and Abx + ORL groups. Values are presented as mean ± SEM. Differences were assessed by Student’s *t*-test and denoted as follows: ^*^p < 0.05, ^**^p < 0.01, and ^***^p < 0.001; ns *p* > 0.05. ^*^In the section of **(A)** body weight means the difference compared with the control group. ^#^*p* < 0.05 and ^##^*p* < 0.01. ^#^Means the difference between ORL and HFD group.

We sought to determine the effect of Orlistat on HFD-fed mice by measuring body weight, food intake, subcutaneous adipose tissue (SAT), and visceral adipose tissue (VAT) weights (Figures 1A,B,D–G). As expected, the HFD significantly increased body weight (*p* < 0.001), relative weight of SAT (*p* < 0.001), and the VAT to body weight ratio in mice (*p* < 0.001). Orlistat treatment significantly inhibited weight gain induced by the HFD (*p* < 0.05). Additionally, Orlistat treatment significantly decreased SAT weight (*p* < 0.01) and VAT weight (*p* < 0.05) in mice. Mice from the ORL group showed greater weight reduction compared to the ORL + antibiotics group, but this difference was not statistically significant (*p* > 0.05). Interestingly, food intake decreased significantly in the ORL + antibiotics group. We further calculated the feed conversion ratio (food intake/weight gain) to assess the relationship between weight gain and food intake (Figure 1C). Here, we found that a higher feed conversion ratio suggests that more food is consumed in order to gain the same amount of weight, which is indicative of a reduced risk of obesity. Compared to the ORL group, the feed conversion ratio in the ORL + Abx group decreased significantly (*p* < 0.001), but there was no significant difference in body weight between the two groups (*p* > 0.05). This suggests that antibiotic treatment suppresses appetite and inhibits fat consumption in mice. Taken together, Orlistat treatment reduced weight gain and fat tissue accumulation induced by the HFD in mice. While antibiotics decreased the feed conversion ratio in the ORL + Abx group, there were no significant differences in body weight and fat tissue weight between the ORL group and the Abx + ORL group.

### 3.2 Antibiotics mitigate Orlistat’s protective effects against hepatic steatosis in HFD mice

**Figure 2:**
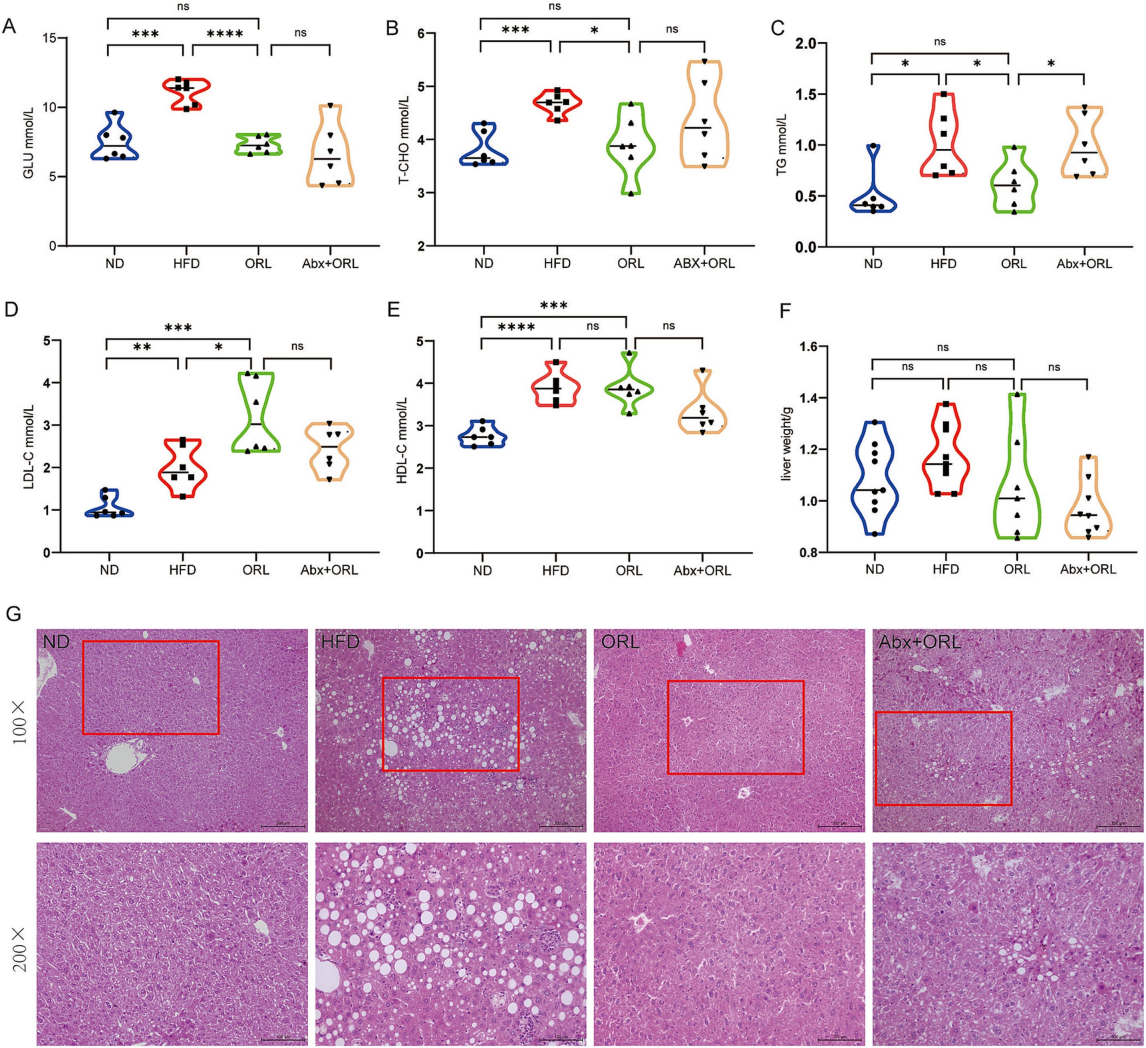
Antibiotic treatment alleviated the inhibitory effect of Orlistat on hepatic steatosis in high-fat diet mice. **(A–E)** Serum concentrations of blood glucose (GLU), total cholesterol (T-CHO), triglycerides (TG), low-density lipoprotein (LDL-C) and (high-density lipoprotein) HDL-C in mice (*n* = 6). **(F)** Mice liver weight in the ND, HFD, ORL and Abx + ORL groups (*n* = 9). **(G)** Liver H&E staining (×200). Values are presented as mean ± SEM. Differences were assessed by Student’s *t*-test and denoted as follows: ^*^p < 0.05, ^**^p < 0.01, and ^***^p < 0.001; ns *p* > 0.05.

We further analyzed serum concentrations of lipid metabolites (Figures 2A–E) and found that the HF diet increased serum levels of GLU, T-CHO, TG, LDL, and HDL (*p* < 0.05). Orlistat administration significantly reduced serum levels of GLU (*p* < 0.001), T-CHO (*p* < 0.05), and TG (*p* < 0.05) compared to the HFD group, but had little effect on the serum level of HDL while improving the serum level of LDL (*p* < 0.05). Mice from the ORL + antibiotics group showed lower LDL and HDL levels compared to the ORL group, but this difference was not statistically significant (*p* > 0.05). Interestingly, TG levels increased significantly in the ORL + antibiotics group. There were no significant differences in GLU and T-CHO serum levels between these groups. In addition, we extracted and weighed the livers of mice in each group and found that there were no statistically significant differences in liver weight among the groups (Figure 2F).

We found the prevalence of steatohepatitis to be 100% in the HFD group. H&E staining revealed normal hepatic ultrastructure in the ND group. Hepatocytes in the ND group showed abundant cytoplasm with centrally located nuclei (Jia et al., 2019). In contrast, hepatocytes in the HFD group exhibited numerous vacuoles (lipid droplets) in their cytoplasm, and they were disordered, deformed, and compressed. In the ORL group, there was minimal liver injury, with most hepatocytes displaying normal ultrastructure, intact cell membranes, and few lipid droplets. Although the degree of liver damage in the ORL + Abx group was less severe than in the HFD group, it was more pronounced compared to the ORL group. The ultrastructure of these hepatocytes was disordered, and lipid droplets were more abundant (Figure 2G). These findings suggest that antibiotic treatment attenuated the inhibitory effect of Orlistat on fatty liver degeneration in HFD mice.

### 3.3 Impact of antibiotic and Orlistat treatments on inflammatory factors and appetite hormone expression in mouse colon

**Figure 3:**
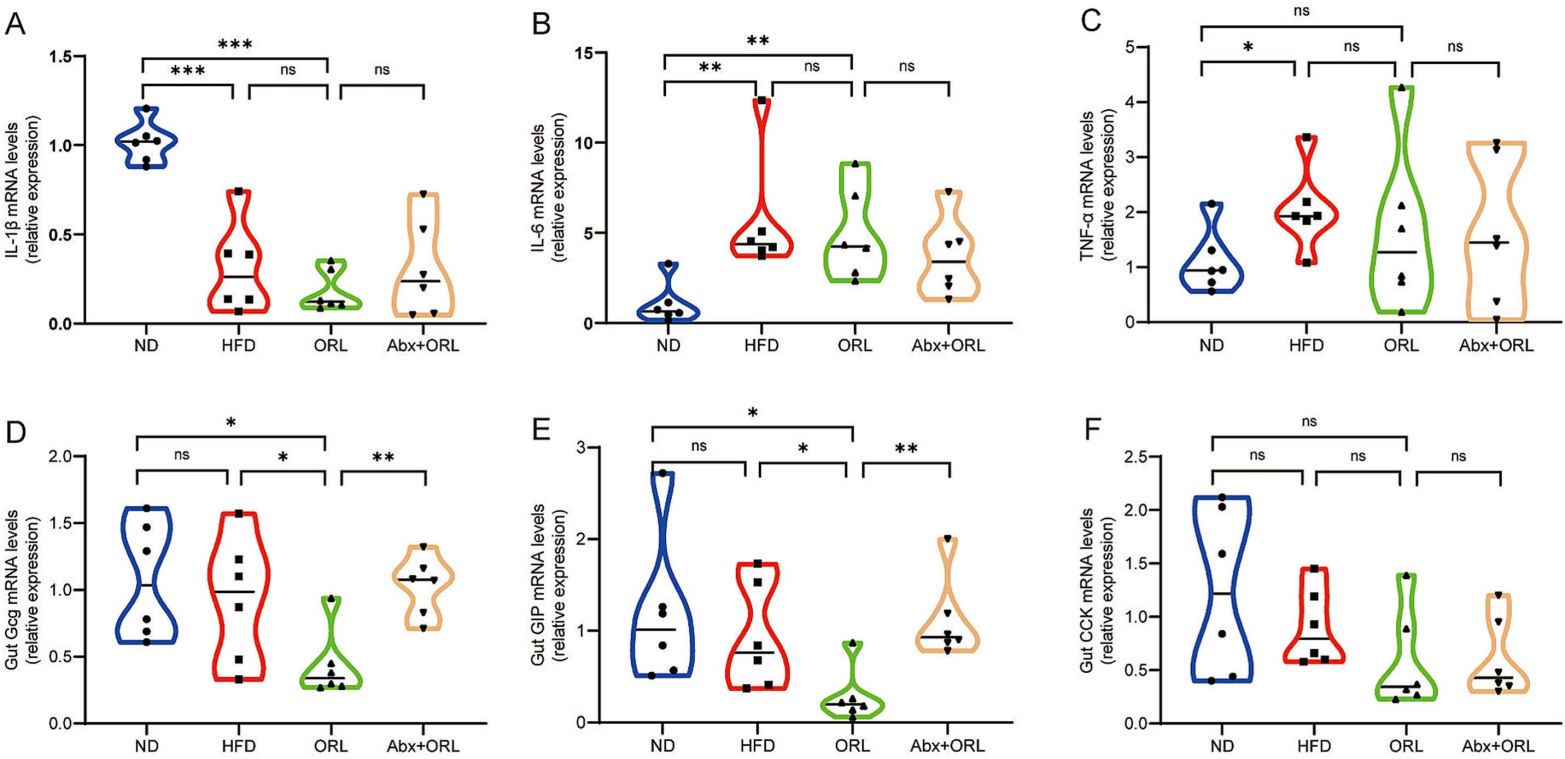
Orlistat treatment significantly decreased the expression of appetite-related hormones *Gcg* and *GIP* in intestinal cells but did not change the gene expression of inflammatory factors in the gut. **(A)** Interleukin-1 beta (*IL-1β*), **(B)** Interleukin-6 (*IL-6*), **(C)** Tumor necrosis factor-alpha (*TNFα*), **(D)** Glucagon (*Gcg*), **(E)** Glucose-dependent insulinotropic polypeptide (*GIP*), and **(F)** Cholecystokinin (*CCK*) mRNA abundance was determined by qRT-PCR analysis, and relative gene expressions were normalized with *GAPDH* (*n* = 6). Values are presented as the mean ± SEM. Differences were assessed by Student’s *t*-test and denoted as follows: ^*^p < 0.05; ^**^p < 0.01; ^***^p < 0.001; ns *p* > 0.05.

Herein, we sought to measure expression levels of the proinflammatory cytokines, IL-1β, IL-6, and TNF-α, which are known to cause inflammation in intestinal tissue. Notably, we found that the expression of IL-1β in the ND group was significantly higher than in the other groups (*p* < 0.001) (Figure 3A). Further, IL-6 and TNF-α expression was consistent with previous findings in the literature (Li et al., 2008). Compared to the ND, the HFD promoted expression of these proinflammatory cytokines (*p* < 0.05). As shown in Figures 3A–C, the expression of IL-1β (Figure 3A), IL-6 (Figure 3B), and TNF-α (Figure 3C) in the HFD group was slightly higher than in the ORL group, but these differences were not statistically significant (*p* > 0.05). Additionally, proinflammatory cytokine expression in the ORL group was generally consistent with the FMT-ORL group. Together, these data show that a HFD increases proinflammatory cytokine expression. Moreover, while Orlistat inhibited fat absorption in mice, it did not reduce proinflammatory cytokine expression, and antibiotic treatment failed to reverse this trend.

We also measured glucagon gene (*Gcg*), glucose-dependent insulinotropic polypeptide (*GIP*), and cholecystokinin (*CCK*) mRNA expression in the colon of all study animals. As shown in Figures 3D–F, *Gcg* (Figure 3D), *GIP* (Figure 3E), and *CCK* (Figure 3F) expression in the HFD group was slightly lower compared to mice in the ND group, but these differences were not statistically significant (*p* > 0.05). Interestingly, when compared to the HFD group, *Gcg* and *GIP* mRNA expression was significantly reduced in the ORL group (*p* < 0.05), while *CCK* expression remained unchanged (*p* > 0.05). Furthermore, when comparing the Abx + ORL group to the ORL group, *Gcg* and *GIP* expression was significantly upregulated (*p* < 0.005), whereas *CCK* expression remained similar (*p* > 0.05). These data may explain the reduced food intake observed in the Abx + ORL group.

### 3.4 ORL alters microbiota composition and function in HFD-fed mice

**Figure 4:**
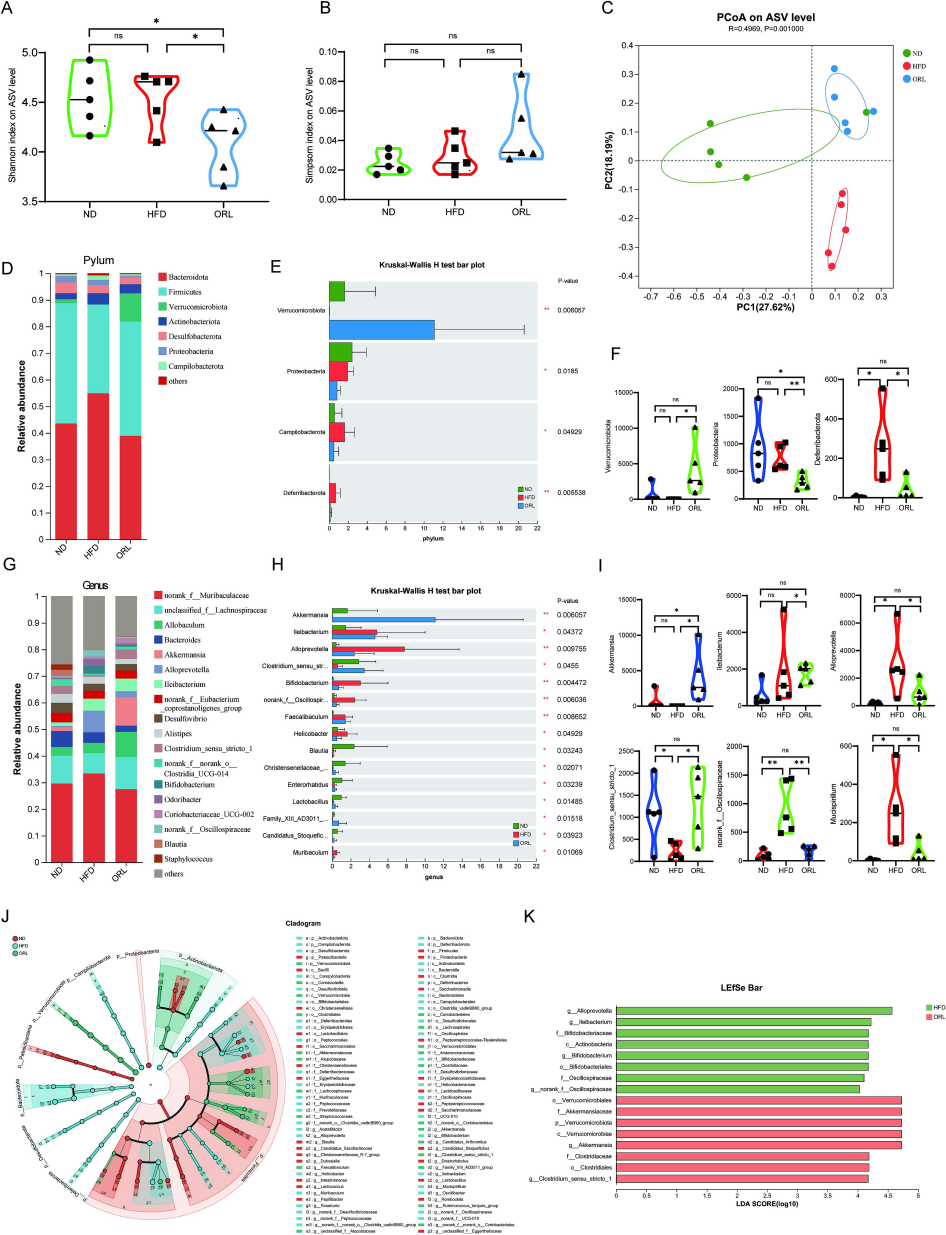
ORL alters microbiota composition and microbiota function in HFD-fed mice. **(A)** Shannon index in α-diversity analysis. **(B)** Simpson index in α-diversity analysis. **(C)** Scatter plot of the principal coordinate analysis (PCoA) score showing the similarity of the 15 bacterial communities based on the Bray–Curtis analysis. Principal components (PCs) 1 and 2 explained 27.62 and 18.19% of the variance, respectively. **(D,F)** Microbiota compositions at the phylum level. **(E)** Phylum level multi-group comparison chart. **(G,I)** Relative abundance of species at the genus level. **(H)** Genus level multi-group comparison chart. **(J)** The taxonomic cladogram and **(K)** a histogram of linear discriminant analysis (LDA) score based on linear discriminant analysis effect size (LEfSe) analysis (LDA >3 was considered to be the differential characteristic taxon). Values are presented as the mean ± SEM. Differences were assessed by Student’s *t*-test and denoted as follows: ^*^p < 0.05, ^**^p < 0.01, and ^***^p < 0.001; ns *p* > 0.05.

The gut microbiota is closely associated with obesity and lipid metabolism. Therefore, we sought to further examine the composition of fecal microbiota samples by performing 16S rRNA gene sequencing of the intestinal microbiota. Shannon and Simpson indices were assessed to evaluate the α diversity of the microbiomes. Compared to the ND and HFD groups, mice in the ORL group exhibited lower microbiota diversity, as indicated by decreased Shannon indices (*p* < 0.05) (Figure 4A). Although the Simpson index increased, this change was not statistically significant (*p* > 0.05) (Figure 4B). Beta diversity analysis, which reflects differences between samples, was also performed. Bray–Curtis distance-based principal coordinate analysis (PCoA) revealed distinct clustering of intestinal microbial communities for each experimental group. Both the HFD and Orlistat interventions induced significant changes in the microbiota community structure. As shown in Figure 4C, PC1 represents the first principal coordinate, accounting for 27.62% of the total variance, while PC2 represents the second principal coordinate, accounting for 18.19% of the total variance. The distinct separation of the ND and HFD groups, along with the significant distance between the ORL and HFD groups in the PCoA plot, suggests that Orlistat consumption induced similar changes in microbial composition (Figure 4C).

Through a comparative analysis of the sequences from the fecal samples, we studied the microbial community structure in Orlistat-treated mice and compared it with the ND and HFD groups. As shown in Figures 4D,F, the gut microbiota at the phylum level primarily consists of *Bacteroidetes*, *Firmicutes*, *Verrucomicrobiota*, and *Actinobacteria*. We employed the Kruskal–Wallis *H* test to evaluate the significance of species abundance differences among multiple sample groups. As illustrated in Figure 4E, the abundance of *Verrucomicrobiota* in the ORL group was higher compared to the ND and HFD groups (*p* < 0.01), while *Proteobacteria* levels were lower (*p* < 0.05). Additionally, *Campylobacterota* levels in the HFD group were higher than those in the ND group (*p* < 0.05), but this trend was reversed with Orlistat supplementation. Relevant studies indicate that *Firmicutes* and *Bacteroidetes* are closely related to glucose and lipid metabolism, as well as bile acid metabolism (Qu et al., 2020). The *Firmicutes* to *Bacteroidetes* ratio (F/B) has become an important indicator for determining obesity (Volokh et al., 2019). A higher F/B ratio suggests greater energy absorption by the intestinal tract, while a lower ratio indicates slower energy absorption, which may help reduce lipid levels (Barczynska et al., 2015). We found that the F/B ratio in the ORL group was slightly higher than in the HFD group; however, this difference was not statistically significant (*p* > 0.05).

The composition of bacterial genera identified in this work is shown in Figures 4G–I, with specific emphasis on genera with a total abundance ratio greater than 0.01. As illustrated in these figures, the abundance of *Alloprevotella* and *Bifidobacterium* in the HFD group was higher than in the ND group, while *Akkermansia*, *Allobaculum*, and *Clostridium_sensu_stricto_1* were lower compared to the ND group. These trends were reversed with Orlistat supplementation, particularly with *Akkermansia* being enriched in the ORL group. Many studies have found that reduced *Akkermansia* abundance is associated with various diseases in mouse models and humans. Further, *Akkermansia* has been shown to improve obesity, type 1 and type 2 diabetes mellitus, hepatic steatosis, intestinal inflammation, and different cancers (including colon cancer and response to immune checkpoint therapies) in mice (Cani et al., 2022). The relative abundance of *Akkermansia* in the ND, HFD, and ORL groups was 1.5, 0.02, and 10.62%, respectively. Overall, these findings indicate considerable changes in the composition of dominant bacteria at the phylum and genus levels in the mouse gut microbiota following Orlistat treatment.

To analyze changes in gut microbiota composition among the different study groups, we used linear discriminant effect size (LEfSe) analysis to identify characteristic taxa in each group (Figures 4J,K). The results indicated that there were eight characteristic taxa in the HFD and ORL groups when the LDA score was >3. In the HFD group, there was a notable increase in *Alloprevotella*, *Ileibacterium*, *Bifidobacterium*, and *norank_f_Oscillospiraceae*. Conversely, the ORL group was enriched in *Akkermansia* and *Clostridium_sensu_stricto_1*.

### 3.5 FMT from Orlistat-treated mice enhances lipid metabolism in HFD mice

We then investigated whether microbiota transplantation from Orlistat-treated mice could alleviate or exacerbate lipid accumulation in HFD mice. HFD mice received either a regular diet or Orlistat by oral gavage for 4 weeks (*n* = 4 for each group). Fecal samples were then collected and transplanted into antibiotic-treated mice (*n* = 9). After 1 week of combined antibiotic treatment, fecal samples were collected from mice in each group, diluted 100-fold, and plated on LB solid medium. The ND group, which did not receive antibiotic treatment, served as the control. The results show a significant reduction in bacterial growth in the FMT-HFD and FMT-ORL groups compared to the ND group, confirming that the antibiotic treatment successfully depleted the majority of gut microbes (Supplementary Figure S1). The microbiota-transplanted mice were subsequently fed a HFD for an additional 2 weeks.

**Figure 5:**
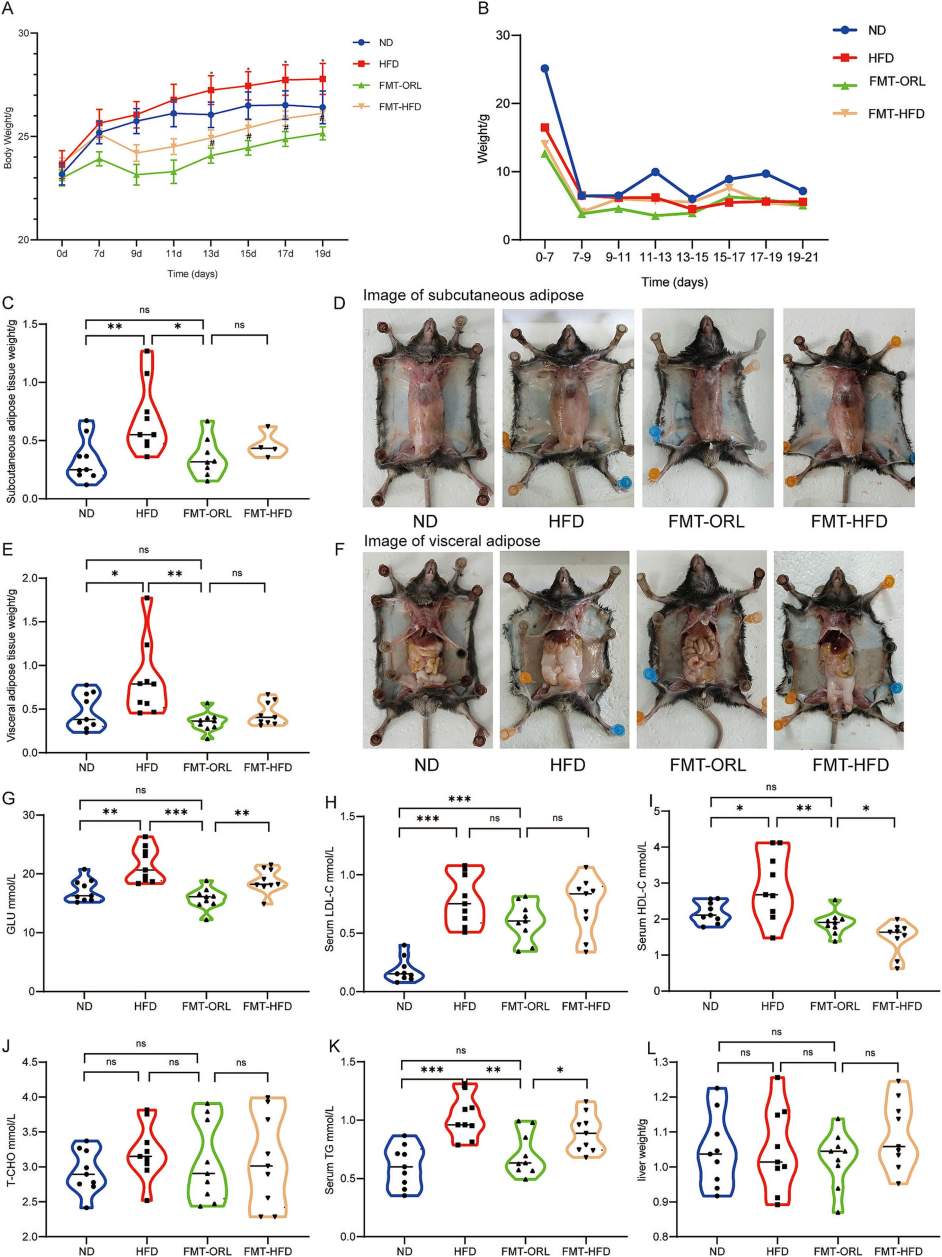
Microbiota transplantation from ORL-treated mice improve lipid metabolism in HF-diet fed mice. Changes in body weight (**A**, x ± s) and average food intake **(B)** in each group of mice (*n* = 9). **(C)** Subcutaneous adipose tissue weight. **(D)** Subcutaneous adipose tissue appearances in animals in the ND, HFD, FMT-ORL and FMT-HFD groups. **(E)** Visceral adipose tissue weight. **(F)** Visceral adipose tissue appearances in animals in the ND, HFD, FMT-ORL and FMT-HFD groups. **(G–K)** Serum concentrations of blood glucose, LDL, HDL, T-CHO and TG in mice (*n* = 9). **(L)** Liver weight in the ND, HFD, FMT-ORL and FMT-HFD groups. Values are presented as the mean ± SEM. Differences were assessed by Student’s *t*-test and denoted as follows: ^*^p < 0.05, ^**^p < 0.01, and ^***^p < 0.001; ns *p* > 0.05. FMT, fecal microbiota transplantation.

As shown in Figure 5A, microbiota transplantation from Orlistat-treated mice inhibited weight gain, which was significantly lower compared to the HFD group (*p* < 0.05) and also lower than the ND group, although this difference was not statistically significant (*p* > 0.05). Interestingly, weight gain in mice in the FMT-HFD group was significantly lower compared to animals in the HFD group (*p* < 0.05) and similar to those in the ND group, but still higher than mice in the FMT-ORL group (*p* > 0.05). There were no statistically significant differences in food intake (Figure 5B). Mice were subsequently sacrificed after 2 weeks of microbiota transplantation. The mass of SAT and VAT in each group is shown in Figures 5C–F. We found that the HFD significantly increased the accumulation of subcutaneous fat (*p* < 0.005) and visceral fat (*p* < 0.05) compared to the ND group. Notably, microbiota transplantation in FMT-ORL treated mice significantly reduced the accumulation of subcutaneous fat (*p* < 0.05) and visceral fat (*p* < 0.01) in high-fat fed mice compared to those in the HFD group. Compared to the FMT-ORL group, the weighed mass of subcutaneous fat and visceral fat in the FMT-HFD group was slightly higher, but these differences were not significant (*p* > 0.05). Furthermore, serum levels of GLU (*p* < 0.01), HDL (*p* < 0.05), and TG (*p* < 0.05) were elevated compared to those in the FMT-ORL group. In addition, there were no significant changes in liver weight (*p* > 0.05) (Figures 5G–L). These results suggest that microbiota transplantation from Orlistat-treated mice reduces fat accumulation and metabolic disturbances in HFD-fed mice.

### 3.6 Microbiota transplantation from Orlistat-treated mice does not influence intestinal appetite hormones or inflammatory factors in HFD mice

**Figure 6:**
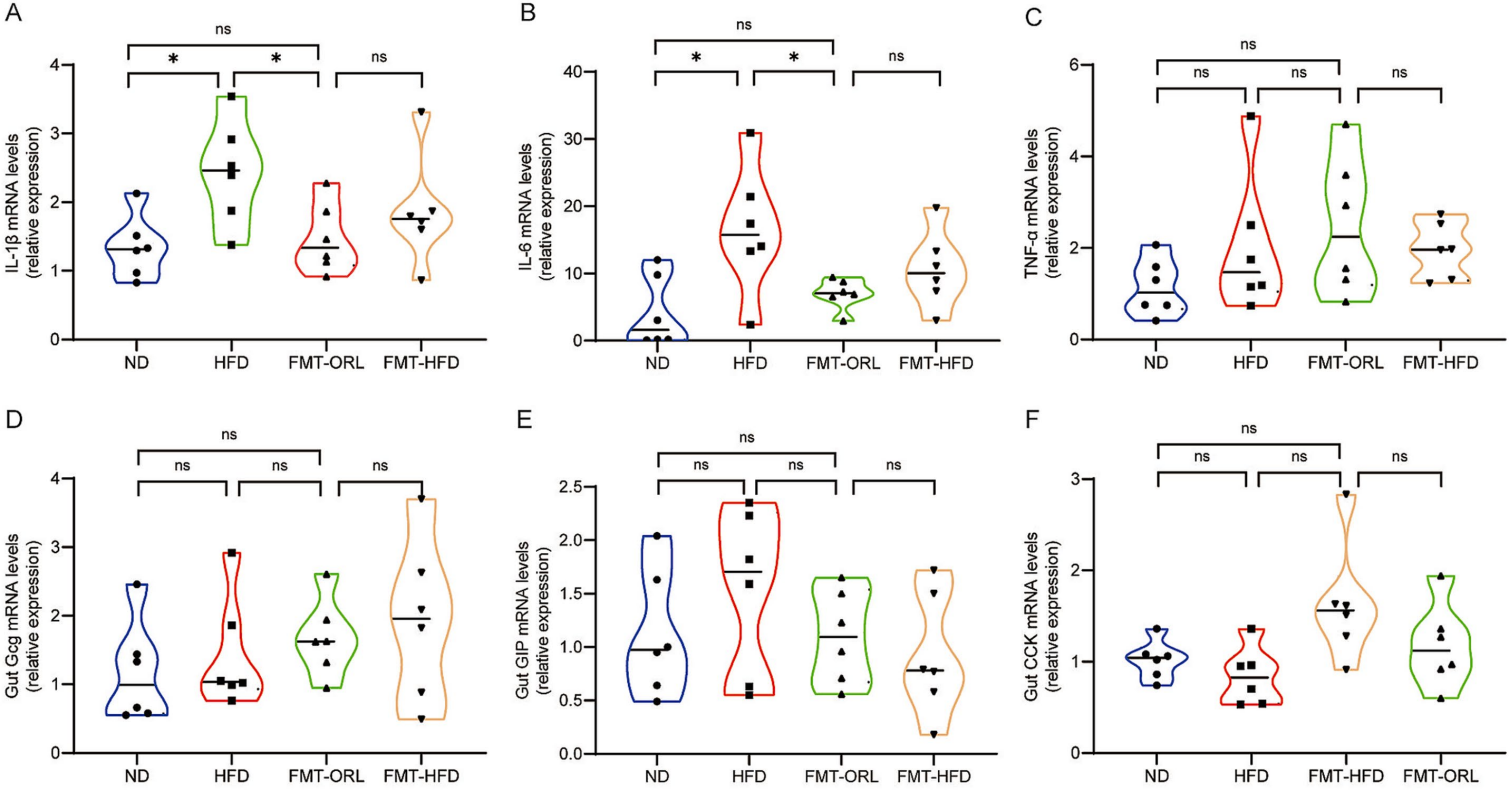
Microbiota transplantation treatment had no effect on the expression of intestinal appetite hormones and inflammatory factors in HFD mice. **(A)** *IL-1β*, **(B)** *IL-6*, **(C)** *TNFα*, **(D)** *Gcg*, **(E)** *GIP*, and **(F)** *CCK* mRNA abundances were determined by qRT-PCR analysis, and relative gene expression was normalized to *GAPDH* (*n* = 6). Values are presented as the mean ± SEM. Differences were assessed by Student’s *t*-test and denoted as follows: ^*^p < 0.05; ^**^p < 0.01; ^***^p < 0.001; ns *p* > 0.05.

To investigate the potential of microbiota transplantation to reduce the expression of intestinal inflammatory factors, we examined the expression levels of IL-1β, IL-6, and TNF-α (Figures 6A–C). We found that, compared to normal mice, mRNA expression of the inflammatory factors *IL-1β* and *IL-6* was significantly increased in HFD-induced obese mice (*p* < 0.05). Compared to the HFD group, *IL-1β* and *IL-6* mRNA expression was significantly reduced in the FMT-ORL group (*p* < 0.05), whereas *TNF-α* expression remained unchanged (*p* > 0.05). Moreover, expression levels of inflammatory factors in the FMT-ORL group were similar to those in the ND group. Interestingly, there were no statistically significant differences in the expression of *IL-1β*, *IL-6*, and *TNF-α* between the FMT-HFD group and the HFD-ORL group (*p* > 0.05).

To investigate whether microbiota transplantation from Orlistat-treated mice can alter the expression of appetite hormones in germ-free recipient mice, we examined the expression levels of the appetite hormones, GIP, Gcg, and CCK, in the FMT recipients (Figures 6D–F). Interestingly, there were no statistically significant differences in the expression of these appetite hormones in the recipient group transplanted with fecal microbiota from Orlistat-treated mice compared to the ND, HFD, and FMT-HFD groups (*p* > 0.05). Additionally, there were no statistically significant differences among the other groups.

### 3.7 Microbiota transplantation from Orlistat-treated mice produces comparable anti-obesity effects in HFD mice

To investigate the effects of intestinal microbiota transplantation on the structure and function of gut microbiota in antibiotic-treated germ-free mice, we conducted 16S rRNA sequencing on the gut microbiota of the FMT recipient mice.

**Figure 7:**
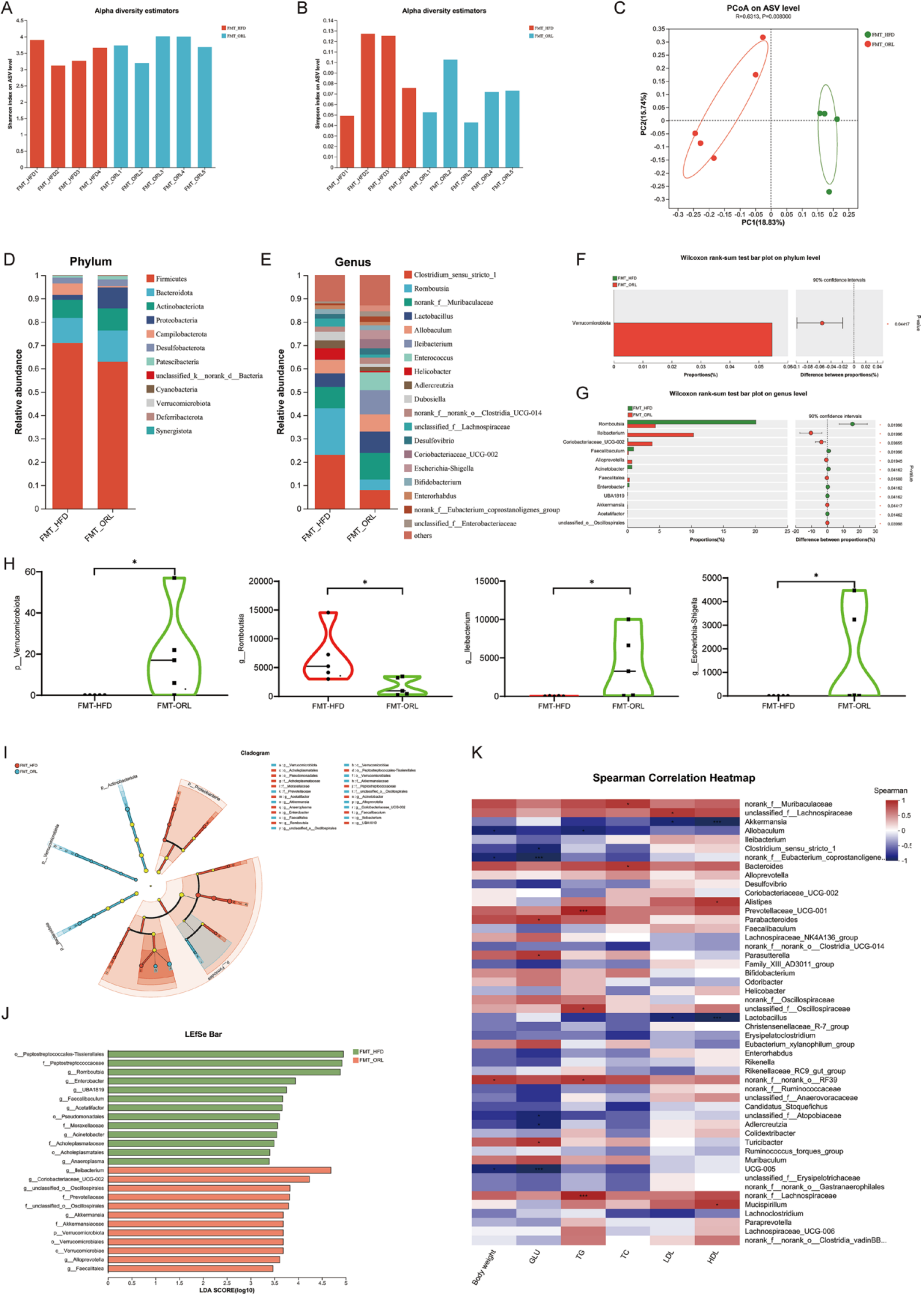
Gut microbiota in response to fecal microbiota transplantation from HF-diet (FMT-HFD) and Orlistat-treated mice (FMT-ORL). **(A)** Shannon index in α-diversity analysis. **(B)** Simpson index in α-diversity analysis. **(C)** Scatter plot of the principal coordinate analysis (PCoA) score showing the similarity of the 10 bacterial communities based on the unweighted_unifrace. Principal components (PCs) 1 and 2 explained 18.83 and 15.74% of the variance, respectively. **(D,E)** Microbiota compositions at the phylum level and genus level. **(F,G)** Phylum and genus level Wilcoxon rank-sum test bar plot. **(H)** Microbiota compositions at the phylum and genus level. **(I)** The taxonomic cladogram and **(J)** histogram of linear discriminant analysis (LDA) score based on linear discriminant analysis effect size (LEfSe) analysis (LDA >2 was considered to be the differential characteristic taxon). **(K)** Spearman correlation analysis was performed to illustrate the correlation between key microorganisms (genus level) regulated by Orlistat and lipid metabolism indicators (body weight, GLU, TG, TC, LDL, HDL). Red indicates positive correlations, while blue indicates negative correlations. Values are presented as the mean ± SEM. Differences were assessed by Student’s *t*-test and denoted as follows: ^*^p < 0.05, ^**^p < 0.01, and ^***^p < 0.001; ns *p* > 0.05.

A comparison of α diversity between the FMT-HFD group and the FMT-ORL group is depicted in Figures 7A,B. The Simpson index for the FMT-HFD group was higher than that for the FMT-ORL group; however, there was no statistically significant difference in the Shannon and Simpson indices between the two groups (*p* > 0.05). Unweighted UniFrac-based PCoA revealed distinct clustering of intestinal microbial communities for both experimental groups (Figure 7C). A significant distance was observed between the FMT-HFD group and the FMT-ORL group, indicating that the microbiota community structure of recipient mice that received fecal bacteria from either the HFD or ORL group underwent notable changes.

Using a comparative analysis of the sequences from the fecal samples, we studied and compared the microbial community structure of the FMT-HFD and FMT-ORL groups. We found that at the phylum level the gut microbiota is primarily composed of *Firmicutes*, *Bacteroidetes*, *Actinobacteria*, and *Proteobacteria* (Figures 7D,F). We used the Wilcoxon rank-sum test to assess species abundance differences among multiple sample groups. As illustrated in Figure 7H, the abundance of *Verrucomicrobiota* in the FMT-ORL group was significantly higher (*p* < 0.05) than in the FMT-HFD group. This finding aligns with previous observations regarding changes in the microbiota composition of mice in the ORL group. The genus-level composition of bacterial types is shown in Figures 7E,G, which highlight those with a total abundance ratio greater than 0.01. As depicted in Figure 7E, the abundance of *Clostridium_sensu_stricto_1* and *Romboutsia* in the FMT-HFD group was higher than observed in the FMT-ORL group, while *norank_f__Muribaculaceae*, *Lactobacillus*, *Ileibacterium*, and *Coriobacteriaceae_UCG-002* were less abundant in the FMT-HFD group compared to the FMT-ORL group. Figure 7G shows that Wilcoxon rank-sum test analysis indicated that *Ileibacterium*, *Coriobacteriaceae_UCG-002*, *Alloprevotella*, and *Akkermansia* were more abundant in the FMT-ORL group than in the FMT-HFD group (*p* < 0.05), while *Romboutsia* was less abundant in the FMT-ORL group (*p* < 0.05). The differences at the genus level between the FMT-ORL and FMT-HFD groups remain largely consistent with those observed earlier. Notably, similar to previous experimental conditions or dietary groups, the abundance of *Akkermansia* and *Allobaculum*, particularly *Akkermansia*, were significantly upregulated in the FMT-ORL group.

To analyze changes in the composition of gut microbiota between the two groups of mice, we used LEfSe analysis to identify characteristic taxa in each group (Figures 7I,J). The results showed that when the LDA score was >3, there were 13 characteristic groups in the FMT-HFD group and 12 characteristic groups in the FMT-ORL group. The FMT-ORL group was particularly rich in the phylum *Verrucomicrobiota*, order Verrucomicrobiales, and genus *Akkermansia*. These findings indicate that mice transplanted with fecal microbiota from the HFD and ORL groups exhibited similar gut microbiota communities when compared to HFD-fed mice and Orlistat-treated mice.

To further analyze the correlation between key microorganisms regulated by Orlistat and lipid metabolism indicators, we performed Spearman correlation analysis and generated a correlation heatmap (Figure 7K). The results revealed a significant negative correlation (*p* < 0.05) between the abundance of *Akkermansia* and the degree of hepatocyte steatosis as well as serum lipid indicators (e.g., TG, TC, LDL). Additionally, the abundance of *Akkermansia* showed a positive correlation with that of *Allobaculum* and *Clostridium_sensu_stricto_1*, suggesting that these microorganisms may play synergistic roles in the improvement of lipid metabolism by Orlistat.

## 4 Discussion

Orlistat, a lipase inhibitor that acts on the gastrointestinal tract, is not absorbed into the bloodstream. As a result of its action, a considerable amount of unabsorbed fat accumulates in the intestine, leading to increased concentrations of TG in the lower gut. This accumulation may potentially alter microbial fermentation in the distal gut. However, the impact of these accumulated fats on the abundance and diversity of the gut microbiome remains unclear. Our studies, along with previous research, have demonstrated that Orlistat treatment significantly reduces obesity and alters gut microbiota in obese mouse models induced by a HFD (Jin et al., 2022; Ke et al., 2020). However, there is currently no direct evidence that the alterations in gut microbiota caused by Orlistat have anti-obesity or obesity-exacerbating effects. To clarify how Orlistat affects the gut microbiota and the role that gut microbiota plays in this process, we propose that the anti-obesity effects of Orlistat are related to changes in gut microbiota. Our research findings suggest that the altered gut microbiota induced by Orlistat has a synergistic effect on its anti-obesity properties, and this capability can be transferred through FMT.

There is an increasing body of evidence to suggest that the gut microbiota is associated with obesity. Several drugs are currently used to treat obesity and type II diabetes, including metformin and acarbose. Metformin can gut microbiota composition by directly affecting bacterial growth and altering the intestinal environment. This drug promotes the growth of bacteria that produce short-chain fatty acids (SCFAs) in the guts of type 2 diabetes mellitus patients, thereby increasing SCFA levels in the colon and improving host metabolism (Wang et al., 2024). Additionally, metformin enhances the protective function of the intestinal mucosal barrier by increasing the abundance of *Akkermansia muciniphila* and goblet cells, leading to a thicker mucus layer that reduces inflammation in the intestine (Wang et al., 2024; Ke et al., 2021). Acarbose is an α-glucosidase inhibitor that slows down the breakdown of starch, thereby reducing blood sugar levels after meals. Similar to Orlistat, this mechanism allows the intestine to accumulate large amounts of undigested starch. A study by Gu et al. (2017) showed that acarbose increases the relative abundances of *Lactobacillus* and *Bifidobacterium* in gut microbiota while depleting *Bacteroides*, thus altering the relative abundance of microbial genes involved in fecal bile acid metabolism. In this study, we used HFD feeding to successfully induce a typical obesity phenotype. Moreover, we showed that Orlistat significantly attenuated HFD-induced obesity. An increased F/B ratio and changes in several bacterial species can promote obesity development in dietary and genetic models in mice (Turnbaugh et al., 2006; Goodman et al., 2011; Turnbaugh et al., 2009). Turnbaugh et al. (2009) suggested that microbial communities with larger F/B ratios are more capable of converting dietary calories into fat. Although the F/B ratio of the HFD group was slightly lower than that of the ND group and the ORL group, this difference was not statistically significant. Notably, the F/B ratios of the ORL group and ND group showed a similar trend, which contradicts findings from previous studies. There is an ongoing debate regarding the association between the F/B ratio and obesity. Some studies have found a positive correlation between the F/B ratio and obesity; however, other studies have reported no modifications to this parameter or even a decreased F/B ratio in obese animals and humans (Jumpertz et al., 2011; Tims et al., 2013; Magne et al., 2020). In this work, we also found that a HFD did not lead to a higher F/B ratio, which may be related to dietary factors. However, further investigation is needed to clarify the association between F/B ratios and obesity.

Consistent with previous studies, Orlistat significantly inhibited weight gain and fat accumulation in HFD-induced mice. It also significantly decreased serum levels of GLU, TC, TG, and low-density lipoprotein cholesterol (LDL-C), thereby improving dyslipidemia and metabolic disorders (Jin et al., 2022; Ke et al., 2020). Additionally, Orlistat significantly improved fatty liver caused by long-term HFD consumption. In this study, we initially explored the role of the gut microbiota in the context of combination treatment with Orlistat and antibiotics. Exposure to antibiotics partially blocked the anti-obesity effects of Orlistat in mice fed a HFD. Compared to the HFD group, serum TG levels in the ORL + Abx group were higher, and the severity of fatty liver was exacerbated. After Orlistat intervention, microbial diversity and the composition of dominant gut bacteria were altered. These results are consistent with other studies on Orlistat intervention in obese mice (Jin et al., 2022; Ke et al., 2020). Specifically, Orlistat modified certain bacterial populations and α-diversity, resulting in increased levels of *Verrucomicrobiota* and *Akkermansia*, but decreased levels of *Alloprevotella* and *Bifidobacterium*. The α-diversity index reflects the richness and homogeneity of gut microbiota, and microbial diversity is often inversely associated with disease development in adulthood. Some studies have indicated that free lipids possess antibacterial properties and can interact directly with host pattern recognition receptors (Cândido et al., 2018; Desbois and Smith, 2010). The decrease in α-diversity observed in the ORL group may be attributed to the high TG environment in the intestine caused by Orlistat, which inhibits the growth of certain intestinal microorganisms. Jin et al. (2022) found that *Verrucomicrobia* were positively correlated with fasting blood GLU and negatively correlated with TC. *Bifidobacterium* is generally considered beneficial bacteria that are inversely associated with obesity; however, different strains can have contradictory effects. For instance, strain M13–4 might increase body weight while strain L66–5 may do the opposite (Yin et al., 2010). Notably, the abundance of *Akkermansia* significantly increased after Orlistat treatment. *Akkermansia* is known to positively impact host health by degrading mucin in the intestine and producing SCFAs, which are important for maintaining gut health, regulating energy metabolism, and improving insulin resistance (Cani et al., 2022; Salem et al., 2024; Derrien et al., 2017). Additionally, *Akkermansia* has been shown to enhance intestinal barrier function and reduce inflammation, potentially helping to prevent obesity-related complications (Ke et al., 2021; Salem et al., 2024). In addition to *Akkermansia*, we observed significant changes in other bacterial populations with potential health benefits following Orlistat treatment. Specifically, there was a notable increase in the relative abundance of *Allobaculum*. This bacterial species is involved in butyric acid production and upregulates the expression of tight junction protein, ZO-1, thereby alleviating LPS-induced increases in intestinal permeability and protecting intestinal barrier function (Zhou et al., 2022). To investigate whether the Orlistat-induced microbial shifts directly contribute to its anti-obesity effects, we transplanted microbiota from Orlistat-treated mice (FMT-ORL) into microbiota-depleted mice alongside microbiota from HFD-fed mice (FMT-HFD). Our findings revealed that compared to the HFD group, the FMT-ORL group exhibited significant reductions in body weight, fat accumulation, serum GLU levels, and TG levels. To mitigate potential biases from gavage and fecal bacteria solution stimulation, we established a positive control group (FMT-HFD), wherein mice were transplanted with fecal bacteria from the HFD group. Notably, blood GLU and TG levels in the FMT-ORL group were significantly lower than those in the FMT-HFD group. Remarkably, the FMT-ORL group displayed a significant increase in *Akkermansia* abundance. Consistent with the ORL group findings, FMT-ORL mice fed on a HFD also exhibited significantly reduced blood GLU and TG levels, suggesting a potential association between *Akkermansia* and Orlistat’s hypoglycemic and lipid-lowering effects. The correlation analysis revealed a significant negative correlation between the abundance of *Akkermansia* and the degree of hepatocyte steatosis as well as serum lipid indicators (e.g., TG, TC, LDL), suggesting that *Akkermansia* may play a crucial role in improving lipid metabolism and alleviating hepatic steatosis. Furthermore, the abundance of *Akkermansia* showed a positive correlation with that of *Allobaculum* and *Clostridium_sensu_stricto_1*, indicating that these microorganisms may act synergistically in the modulation of gut microbiota by Orlistat, collectively contributing to the improvement of lipid metabolism. Intriguingly, changes in gut microbiota communities resulting from FMT-HFD and FMT-ORL were similar to those observed in HFD and ORL mice. This indicates that microbiota transplantation from Orlistat-treated mice exerts a similar anti-obesity effect in HFD-fed mice, suggesting a causal role for microbiota in lipid metabolism.

In this work, we have revealed an intriguing phenomenon: the administration of Orlistat to mice fed on a HFD significantly increased their food consumption while significantly reducing their overall body weight. This finding aligns with previous studies (Ellrichmann et al., 2008; Enç et al., 2009), which demonstrated that Orlistat can effectively lower serum levels of appetite-regulating hormones, including glucagon-like peptide-1 (GLP-1), CCK, peptide YY (PYY), and GIP. Callahan et al. (2016) showed that Orlistat increases appetite and attenuates postprandial concentrations of GLP-1, CCK, PYY. Similarly, other studies have also shown that Orlistat accelerates gastric emptying and reduces GIP release in healthy subjects (Bolyen et al., 2019). GIP is an incretin hormone that belongs to the glucagon superfamily; it acts as a potent stimulator of insulin secretion and inhibits gastric acid secretion (Jia et al., 2019; Qu et al., 2020). The GIP protein, encoded by *Gcg*, is processed to generate glucagon and two other glucagon-like peptides, GLP-1 and GLP-2. Glucagon stimulates gluconeogenesis, glycogenolysis, and lipolysis, while GLP-1 induces insulin secretion, suppresses glucagon secretion, and inhibits feeding (Jia et al., 2019; Qu et al., 2020). To further explore this phenomenon, we conducted qRT-PCR analysis on the intestinal tissues of mice treated with Orlistat. Our analysis revealed a decrease in mRNA gene expression levels of appetite-regulated hormones such as *Gcg*, *GIP*, and *CCK* in the small intestine, consistent with previous research. However, in the group that received a combination treatment of antibiotics and Orlistat, we observed an increase in the gene expression of *Gcg* and *GIP* in small intestinal tissue. This observation may explain the decreased food intake seen in the ORL + Abx group. Additionally, we examined the expression of appetite hormone genes in the small intestinal tissues of mice that underwent FMT and received Orlistat. Intriguingly, the side effects of Orlistat did not appear to transfer with FMT; the expression of appetite hormone-related genes in the small intestine of FMT recipient mice treated with Orlistat remained unchanged. Some studies have shown that SCFAs are directly involved in hormone secretion, stimulating L-intestinal endocrine cells to release GLP-1 and PYY, thereby suppressing appetite (Chambers et al., 2015; Gribble and Reimann, 2019). Therefore, a possible mechanism by which Orlistat inhibits appetite hormone production may be related to its inhibition of SCFA-producing bacteria; a decrease in SCFA content could lead to suppressed appetite hormone secretion. However, we did not observe changes in appetite hormone expression in the fecal microbiota of mice, which may indicate that maintaining low levels of appetite hormones requires ongoing Orlistat treatment. This finding suggests that Orlistat affects the expression of appetite hormones through a gut microbiota mechanism. The precise underlying mechanism requires further investigation and elucidation.

Metabolic endotoxemia resulting from dysbiotic gut microbiota. Further, impaired gut barrier integrity plays a central role in the pathogenesis of chronic low-grade inflammation, which is a key factor in obesity and its associated health complications (Cani et al., 2008; Caesar et al., 2015). In our study, we found that Orlistat did not alter the expression of inflammatory factors in mice fed a HFD. The expression levels of inflammatory factors IL-1β and IL-6 were significantly elevated in the HFD and ORL groups, with no difference between the two. Furthermore, the addition of antibiotics did not affect the increased expression of inflammatory factors in the Abx + ORL group of mice. Some studies have shown that saturated fatty acids can directly interact with host pattern recognition receptors, activating Toll-like receptor 4 (TLR4) and TLR2, which promotes the synthesis of inflammatory factors (Huang et al., 2012). Interestingly, Orlistat treatment resulted in a significant accumulation of TG in the intestines of mice in the ORL group; however, this did not lead to an increase in the expression of inflammatory factors. We speculate that this may be related to the increased abundance of *Akkermansia muciniphila*, which has been shown to enhance intestinal barrier function and reduce inflammatory responses (Ke et al., 2021; Salem et al., 2024), thereby counteracting the proinflammatory effects of TG.

In summary, Orlistat improves lipid metabolism, reduces fat accumulation, and alters the composition of the intestinal microbiota, particularly by significantly increasing the abundance of *Akkermansia* bacteria. Notably, this regulatory effect can be transferred to other individuals through FMT. However, antibiotic-induced depletion of microbiota significantly weakens Orlistat’s ability to improve lipid metabolism; this effect can be restored through intestinal microbiota transplantation. It is important to note that some side effects of Orlistat, such as the decreased expression of appetite hormone-related genes, do not transfer with fecal transplantation. This further emphasizes the crucial role of intestinal microbiota in mechanistic drug action. Through in-depth analysis, we found a significant negative correlation between the increase in *Akkermansia* abundance and the degree of hepatocyte steatosis and serum lipid indicators. Although this trend is clearly observed, the specific underlying mechanisms by which *Akkermansia muciniphila* intervenes and improves metabolic disorders still requires further research in the future. In conclusion, this study not only analyzes how Orlistat alleviates lipid metabolism disorders in HFD mice by adjusting the intestinal microbiota but also provides new perspectives for a comprehensive understanding of Orlistat’s anti-obesity mechanisms.

## Data Availability

The original contributions presented in the study are publicly available. This data can be found here: https://www.ncbi.nlm.nih.gov/sra, accession number PRJNA1130423.

## Footnotes

^1^ www.majorbio.com
