# Supplementary figures and images for: Orlistat ameliorates lipid dysmetabolism in high-fat diet-induced mice via gut microbiota modulation
Source: Front Microbiol. 2025 Feb 6;16:1480500. doi: 10.3389/fmicb.2025.1480500 (PMC11839628; doi:10.3389/fmicb.2025.1480500)

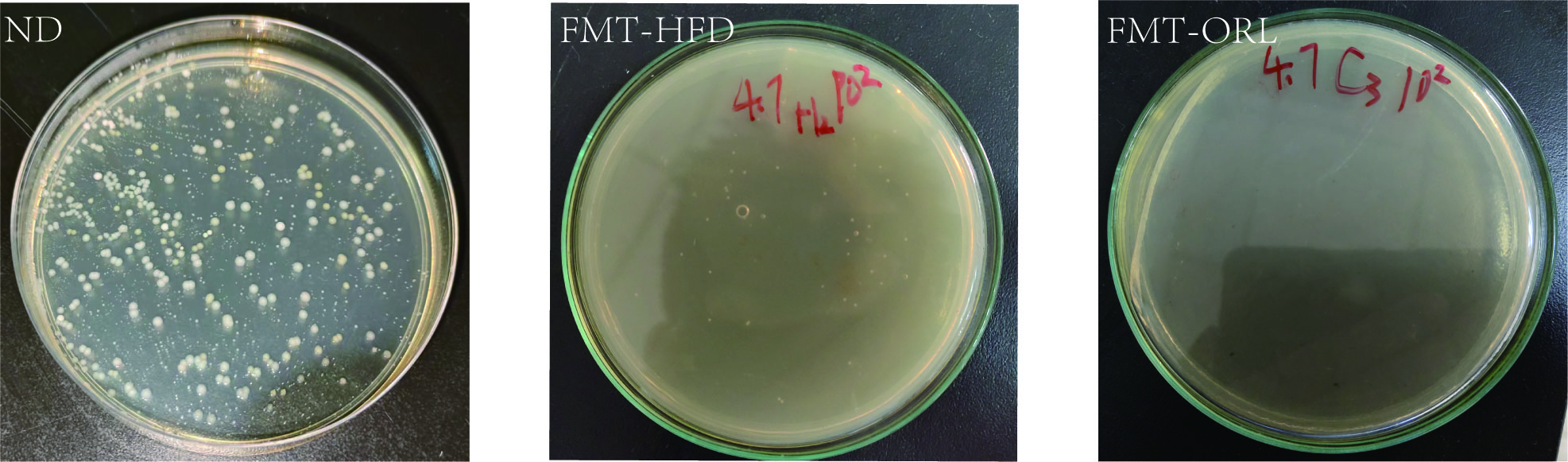

Supplement: Supplementary file 1 [file Image_1.jpeg]
